# Mitochondria Are the Target Organelle of Differentiation-Inducing Factor-3, an Anti-Tumor Agent Isolated from *Dictyostelium Discoideum*

**DOI:** 10.1371/journal.pone.0072118

**Published:** 2013-08-15

**Authors:** Yuzuru Kubohara, Haruhisa Kikuchi, Yusuke Matsuo, Yoshiteru Oshima, Yoshimi Homma

**Affiliations:** 1 Department of Molecular and Cellular Biology, Institute for Molecular and Cellular Regulation, Gunma University, Maebashi, Japan; 2 Laboratory of Natural Product Chemistry, Tohoku University Graduate School of Pharmaceutical Sciences, Aoba-yama, Aoba-ku, Sendai, Japan; 3 Department of Biomolecular Science, Institute of Biomedical Sciences, Fukushima Medical University School of Medicine, Fukushima, Japan; AMS Biotechnology, United Kingdom

## Abstract

Differentiation-inducing factor-3 (DIF-3), found in the cellular slime mold *Dictyostelium discoideum,* and its derivatives such as butoxy-DIF-3 (Bu-DIF-3) are potent anti-tumor agents. However, the precise mechanisms underlying the actions of DIF-3 remain to be elucidated. In this study, we synthesized a green fluorescent derivative of DIF-3, BODIPY-DIF-3, and a control fluorescent compound, Bu-BODIPY (butyl-BODIPY), and investigated how DIF-like molecules behave in human cervical cancer HeLa cells by using both fluorescence and electron microscopy. BODIPY-DIF-3 at 5–20 µ M suppressed cell growth in a dose-dependent manner, whereas Bu-BODIPY had minimal effect on cell growth. When cells were incubated with BODIPY-DIF-3 at 20 µM, it penetrated cell membranes within 0.5 h and localized mainly in mitochondria, while Bu-BODIPY did not stain the cells. Exposure of cells for 1–3 days to DIF-3, Bu-DIF-3, BODIPY-DIF-3, or CCCP (a mitochondrial uncoupler) induced substantial mitochondrial swelling, suppressing cell growth. When added to isolated mitochondria, DIF-3, Bu-DIF-3, and BOIDPY-DIF-3, like CCCP, dose-dependently promoted the rate of oxygen consumption, but Bu-BODIPY did not. Our results suggest that these bioactive DIF-like molecules suppress cell growth, at least in part, by disturbing mitochondrial activity. This is the first report showing the cellular localization and behavior of DIF-like molecules in mammalian tumor cells.

## Introduction

**Figure 1:**
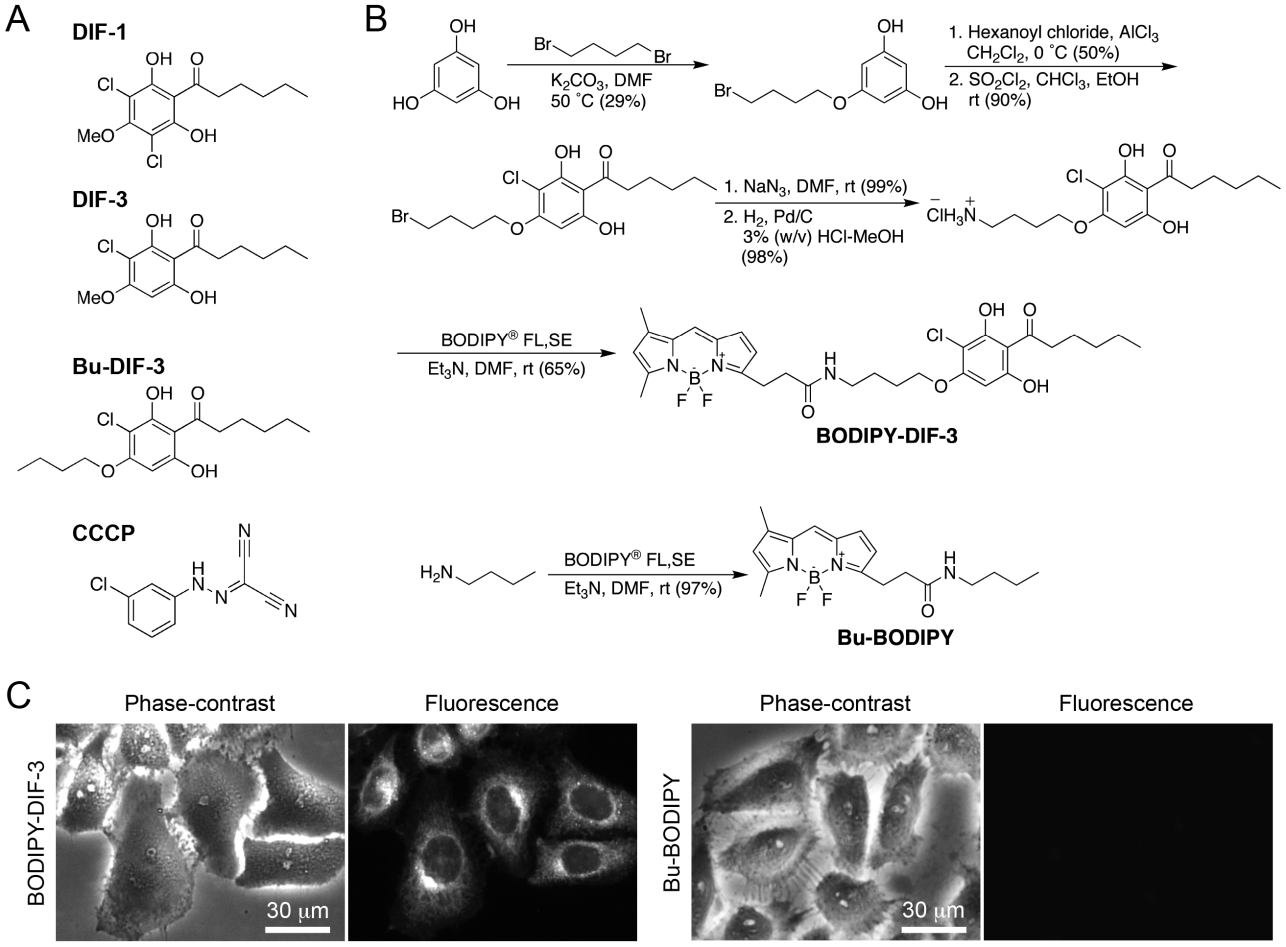
Chemical structures of DIF-like molecules, synthesis of BODIPY-conjugated compounds, and cellular localization of BODIPY-conjugated compounds in HeLa cells. (A) Chemical structures of DIF-1, DIF-3, Bu-DIF-3, and CCCP. DIF-1 [1-(3,5-dichloro-2,6-dihydroxy-4-methoxyphenyl)hexan-1-one] and DIF-3 [1-(3-chloro-2,6-dihydroxy-4-methoxyphenyl)hexan-1-one] are endogenous factors in *D. discoideum*. Bu-DIF-3 (butoxy-DIF-3) [1-(3-chloro-2,6-dihydroxy-4-butoxyphenyl)hexan-1-one] is an artificially designed derivative of DIF-3. The order of compounds with highest to lowest anti-proliferative activity was established as Bu-DIF-3> DIF-3> DIF-1. CCCP (carbonyl cyanide *m*-chlorophenylhydrazone) is a mitochondrial uncoupler. (B) Scheme for synthesis of the BODIPY-conjugated compounds. BODIPY-DIF-3 and Bu-BODIPY were synthesized as described in the Methods section. (C) Cells were incubated for 0.5 h with 20 µM of Bu-BODIPY or BODIPY-DIF-3, washed free of them, and observed microscopically.

The cellular slime mold *Dictyostelium discoideum* is a soil micro-organism that forms a fruiting body consisting of spores and a multicellular stalk at the end of its life cycle. Differentiation-inducing factor-1 (DIF-1) (Figure 1A) is a putative morphogen that induces stalk cell differentiation in *D. discoideum* [1]. DIF-3 (Figure 1A) is the first metabolite produced during the degradation of DIF-1 and has virtually no activity in the induction of stalk cell differentiation [2], [3]. Recently, it was shown that DIF-1 functions not only as a differentiation-inducing factor but also as a modulator of chemotactic movement in *D. discoideum* cells [4]. However, the precise mechanisms underlying the actions of DIF-1 remain to be elucidated, and there have been no receptor(s) identified for DIF-1.

In addition to their physiological activity in *D. discoideum*, DIF-1 and DIF-3 possess anti-tumor activity by suppressing cell growth and, in some cases, by inducing or promoting the differentiation of de-differentiated tumor cells in vitro [5]–[11]. Interestingly, DIF-3 is more active than DIF-1 in suppressing cell growth and inducing erythroid differentiation in human K562 leukemia cells [8], [12]. In a previous study, we showed that several chemically synthesized derivatives of DIF-3 with a modification at the methoxy group, such as butoxy-DIF-3 (Bu-DIF-3) (Figure 1A), have a potent anti-proliferative effect in K562 cells [13]. These findings suggest that derivatives of DIF-3 are potential candidates for development as novel anti-cancer drugs. The mechanisms underlying the actions of DIF-like molecules have been investigated by using several approaches [6]–[11], [14] but remain to be elucidated.

In the present study, we synthesized a green fluorescent derivative of DIF-3, BODIPY-DIF-3, and a control fluorescent compound, Bu-BODIPY (Figure 1B), to investigate the cellular localization, function, and target proteins of DIF-like molecules in mammalian cells using mainly HeLa cells as a model system. We show here that BODIPY-DIF-3 localized mainly to the mitochondria and that BODIPY-DIF-3, as well as DIF-3 and Bu-DIF-3, suppressed cell growth, whereas Bu-BODIPY had no effect. We also show that DIF-3, Bu-DIF-3, and BODIPY-DIF-3 have potential as potent mitochondrial uncouplers to induce morphological change and dysfunction of mitochondria. In summary, our results suggest that DIF-like molecules suppress cell growth, at least in part, by disturbing mitochondrial activity.

## Results

### Cellular Localization of BODIPY-DIF-3

**Figure 2:**
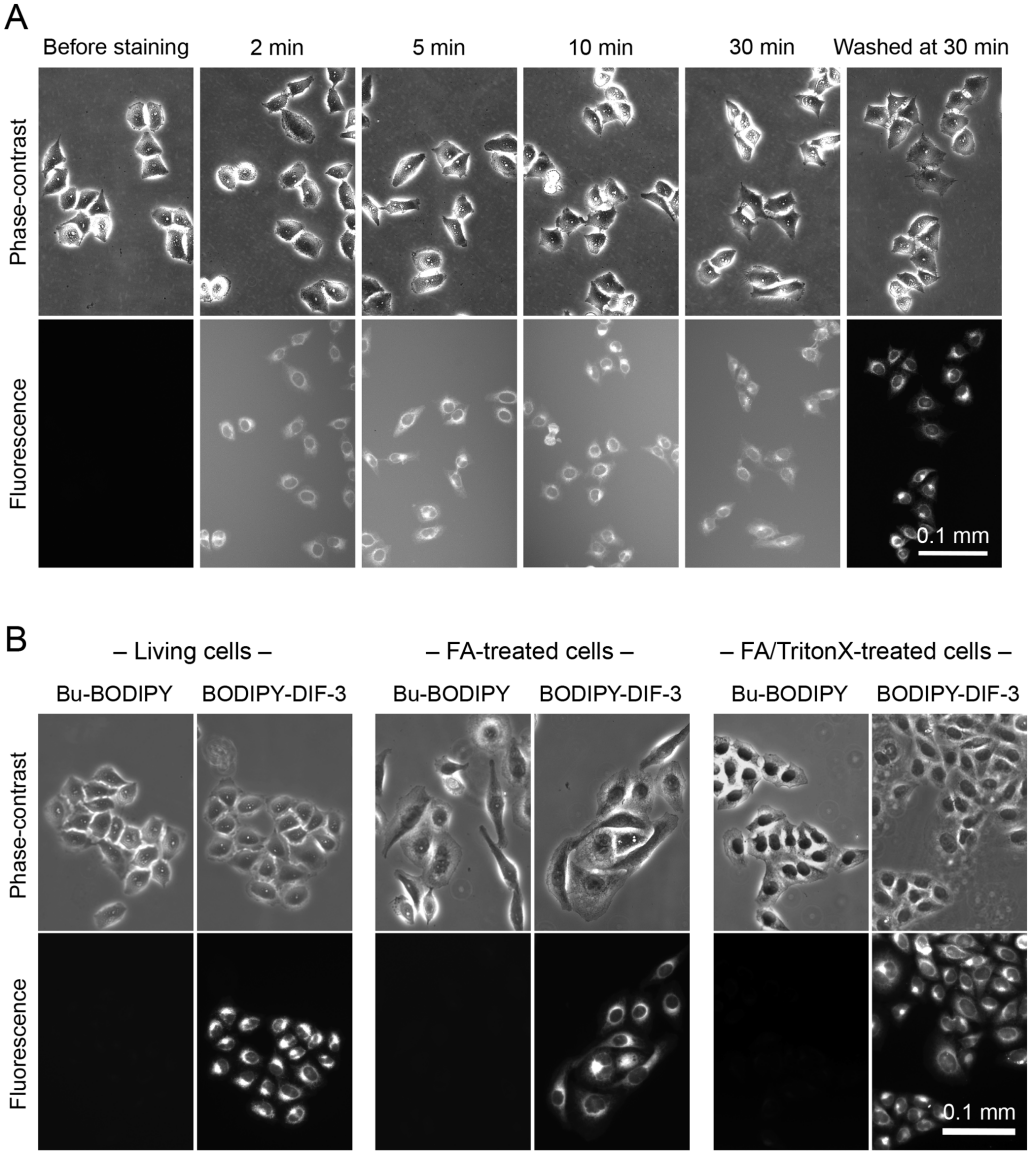
Membrane permeability and localization of BODIPY-DIF-3 and Bu-BODIPY in living, formalin-fixed, and detergent-treated HeLa cells. (A) Cells were incubated with 20 µM BODIPY-DIF-3 and observed microscopically at the indicated time points. After 30 min of incubation, cells were washed free of the additive and observed again. BODIPY-DIF-3 rapidly penetrated the cell membrane within 2 min of exposure, reaching a maximal level within 30 min. (B) Living cells, formalin (FA)-fixed cells, and FA-fixed and TritonX-100-treated cells were incubated for 0.5 h with 20 µM of Bu-BODIPY or BODIPY-DIF-3, washed free of the additives, and observed microscopically. All cell samples stained well with BODIPY-DIF-3 but not with Bu-BODIPY.

To elucidate the cellular localization and function of DIF-like molecules in mammalian cells, we first designed and synthesized a fluorescent derivative of DIF-3, BODIPY-DIF-3 (Figure 1B), in accordance with our structure–activity relationship assay; it was expected that modification of the methoxy region of DIF-3 with BODIPY would not reduce its biological activity [13]. We also synthesized a control fluorescent compound, Bu-BODIPY (butyl-BODIPY) (Figure 1B). Cellular localizations of the two fluorescent compounds were then investigated in HeLa cells. BODIPY-DIF-3 at 20 µM was rapidly incorporated into the cells within several minutes of exposure and a fluorescence signal was detected within 30 min (Figure 2A). Moreover, BODIPY-DIF-3 was found to accumulate slightly in the plasma membrane but mainly in the intracellular membranes of some organelles (Figure 1C). Cells treated with Bu-BODIPY at the same concentration did not show any notable staining (Figure 1C). Essentially the same cellular BODIPY-DIF-3 localization was observed in mouse LM8 osteosarcoma (Figure S1B) and mouse 3T3-L1 fibroblast cells (Figure S2B).

It is noteworthy that, as well as living HeLa cells, formalin-treated and formalin/Triton X-treated (permeabilized) cells stained with BODIPY-DIF-3, but none of them showed any notable staining with Bu-BODIPY (Figure 2B). Since the DIF-3-region of BODIPY-DIF-3 appears to be responsible for the cellular localization of this molecule, it is probable that imaging analysis with BODIPY-DIF-3 would reflect the true cellular localization of DIF-3-like molecules such as DIF-3 and Bu-DIF-3.

### Effect of DIF-3 Derivatives on Cell Growth and Expression of Cyclin D

**Figure 3:**
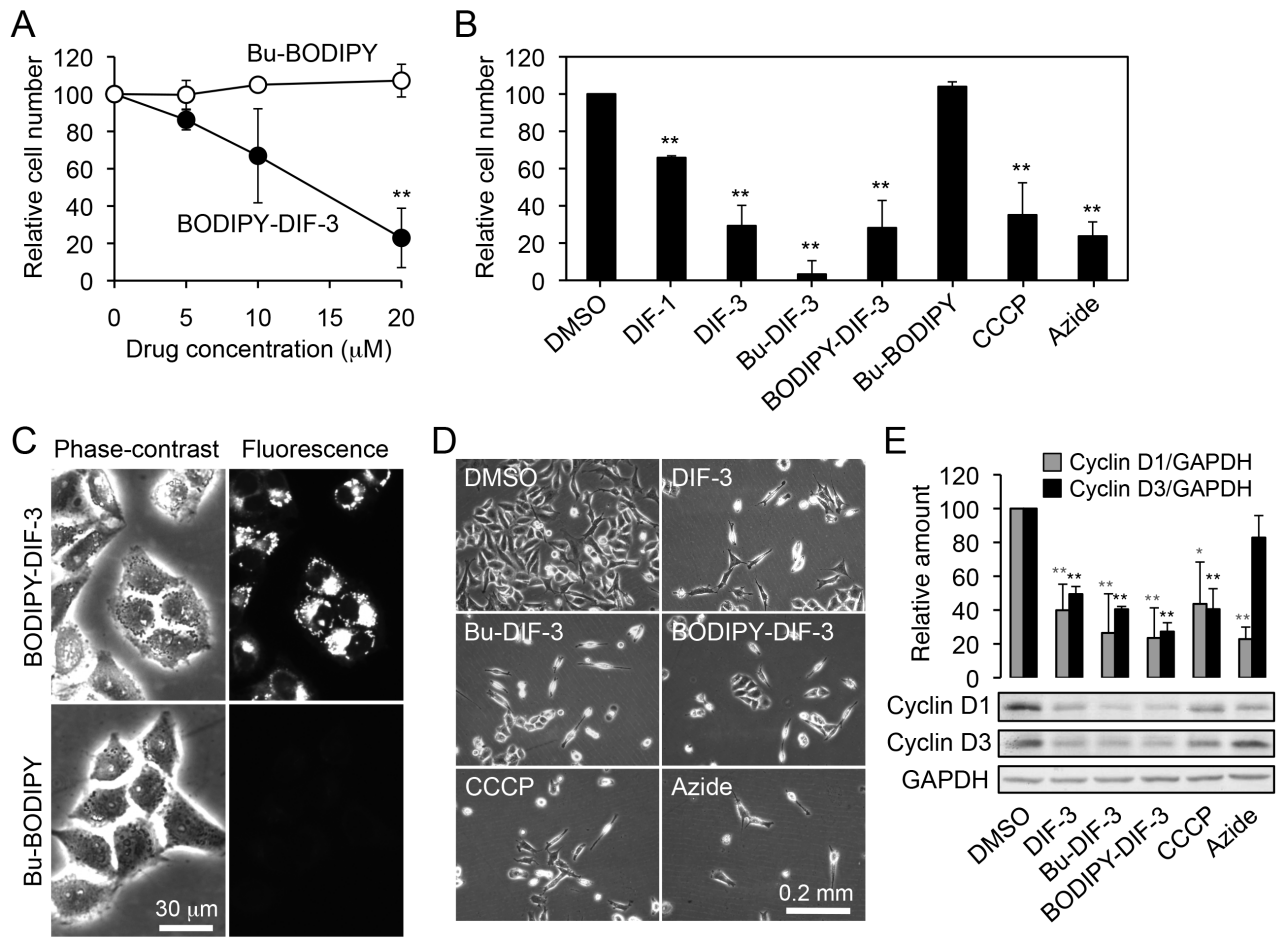
Effects of DIF-like molecules and mitochondrial poisons on cell growth and morphology in HeLa cells. (A) Cells were incubated for 3 days with 0.2% DMSO (vehicle; open circles), 5–20 µM of Bu-BODIPY (open circles), or BODIPY-DIF-3 (closed circles) and relative cell number was assessed by the use of Alamar blue (a cell-number indicator). The mean values and SD (bars) of four independent experiments are presented. **, *P*<0.01 versus DMSO control (Concentration, 0). (B) Cells were incubated for 3 days with 0.2% DMSO, 20 µM of DIF-like molecules, 20 µM of BODIPY-coupled compounds, 10 µM CCCP, or 0.01% NaN_3_ (Azide), and relative cell number was assessed by the use of Alamar blue. The mean values and SD (bars) of five independent experiments are presented. **, *P*<0.01 versus DMSO control. (C) Cells were incubated for 3 days with 20 µM of Bu-BODIPY or BODIPY-DIF-3, washed free of them, and observed microscopically. Note that BODIPY-DIF-3 stained some organelles, which appeared to be disturbed as compared to those at Day 0 (Figure 1C). (D) Cells were incubated for 3 days with 0.2% DMSO, 20 µM DIF-3, 5 µM Bu-DIF-3, 20 µM BDOIPY-DIF-3, or 10 µM CCCP, and observed by using a phase-contrast microscope. (E) Cells were incubated for 20 h with 0.2% DMSO, 20 µM DIF-3, 5–7.5 µM Bu-DIF-3, 20 µM BDOIPY-DIF-3, or 10 µM CCCP, and the indicated cell proteins were analyzed by Western blotting. Relative amounts of cyclin D1and D3 were normalized with GAPDH (glyceraldehyde-3-phosphate dehydrogenase), and the mean values and SD (bars) of three independent experiments are shown together with representative images of the blots. Note that cyclin D2 was not well detected in our system. *, *P*<0.05; **, *P*<0.01 versus DMSO control.

We then examined and compared the effects of DIF-like molecules and the fluorescent compounds on cell growth in HeLa cells (Figure 3). As expected, cell growth was significantly suppressed by the positive control drugs DIF-1, DIF-3, and Bu-DIF-3 at 20 µM (Figure 3B). Cell growth was hardly affected by Bu-BODIPY at up to 20 µM (Figure 3A, B) but was suppressed in a dose-dependent manner with BODIPY-DIF-3 at 5–20 µM (Figure 3A, B). By comparison, cells were alive and appeared unaffected even after 3 days of incubation with the BODIPY-conjugated compounds (Figure 3C, D). However, distinct staining of intracellular structures was apparent after incubation of cells for 3 days with 20 µM BODIPY-DIF-3 (Figure 3C). BODIPY-DIF-3 dose-dependently suppressed cell growth in LM8 and 3T3-L1 cells (Figure S1A, S2A) and also disturbed their intracellular structures at Day 3 (Figure S1B, S2B).

Since DIF-1 and DIF-3 has been shown to decrease the expression of cyclin D (mainly, cyclin D1), thereby suppressing cell growth in HeLa and other cell lines [10]–[12], [15], we compared the effects of DIF-like molecules on cyclin D expression in HeLa cells. Western blot analysis revealed that BODIPY-DIF-3 (20 µM), as well as DIF-3 (20 µM) and Bu-DIF-3 (5–7.5 µM), significantly decreased the expression of cyclin D1 and cyclin D3 after 20 h incubation with the compounds (Figure 3E).

Thus, BODIPY-DIF-3 is not the most potent anti-proliferative compound among the DIF-like molecules, but it does possess the same biological activities as DIF-3 and Bu-DIF-3, thus confirming that BODIPY-DIF-3 would be a suitable probe to investigate the behavior and cellular localization of DIF-like molecules in mammalian cells.

### Effects of BODIPY-DIF-3 on Mitochondrial Morphology

**Figure 4:**
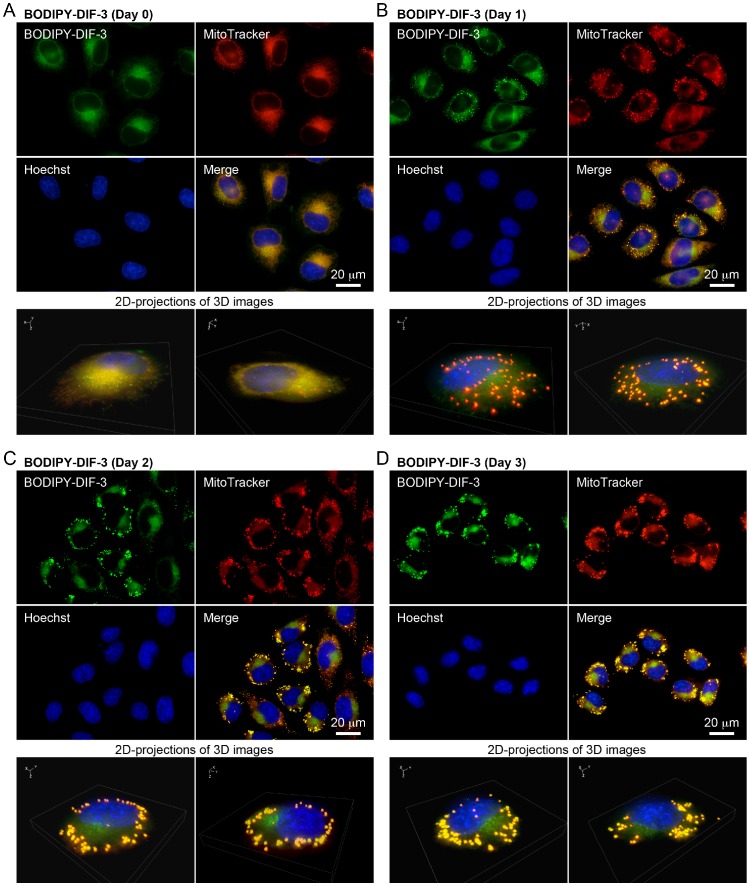
Cellular localization of BODIPY-DIF-3 in HeLa cells. (A) Cells were incubated for 0.5 h with BODIPY-DIF-3 (20 µM), Hoechst (0.1 µg/ml) and MitoTracker (0.1 µM), washed free of the additives, and observed by using a high-magnification fluorescence microscope. (B–D) Cells were incubated for 1–3 days with BODIPY-DIF-3 (20 µM) and then for 0.5 h with Hoechst (0.1 µg/ml) and MitoTracker (0.1 µM). Cells were washed free of the additives and observed by using a high-magnification fluorescence microscope. Three-dimensional (3D) images were constructed from z-stacked 2D images, and two representative 2D-projections of the 3D images are shown (A–D). BODIPY-DIF-3 and MitoTracker co-localized to the mitochondria.

We then performed a detailed analysis of the intracellular localization of BODIPY-DIF-3 by using a high-magnification fluorescence microscope and several fluorescent probes specific for organelles and found that BODIPY-DIF-3 localized mainly in mitochondria. At the start of incubation with the drugs (Day 0), BODIPY-DIF-3 and MitoTracker co-localized to the mitochondria in HeLa cells (Figure 4A). Exposure of the cells to 20 µM BODIPY-DIF-3 caused many mitochondria to swell in most cells within 1 day; these mitochondria stained with BODIPY-DIF-3 and MitoTracker (Figure 4B). By Day 3, further mitochondrial enlargement was observed in almost all of the cells exposed to BODIPY-DIF-3 (Figure 4C, D). Similar results were obtained with LM8 and 3T3-L1 cells (Figure S1C, D, S2C, D). Note that LM8 and HeLa (tumor) cells were more sensitive to all the DIF-3 derivatives (DIF-3, Bu-DIF-3, and BODIPY-DIF-3) than 3T3-L1 (non-transformed) cells (Table 1).

**Table 1:** Comparison of the effects of DIF-like molecules and CCCP.

		IC_50_ (µM) vs. Cell growth			PC_100_ (µM) for	
Compound	M.W.	HeLa	LM8	3T3-L1	Mito. O_2_ consumption	ClogP
DIF-3	272.7	14.5	15.5	>20	12	3.79
Bu-DIF-3	314.8	3.2	2.0	4.3	1.7	5.38
BODIPY-DIF-3	603.9	13.2	12.2	20	25	7.02
Bu-BODIPY	347.2	>20	>20	>20	>100	4.45
CCCP	204.6	5.2	6.8	7.5	0.43	3.40

Footnotes. IC_50_ (50% inhibitory concentration) values versus cell growth of HeLa, LM8, and 3T3-L1 cells were determined from drug dose–dependent growth assays. The PC_100_ (100% promoting concentration) values for the control rate of mitochondrial (Mito.) O_2_ consumption were calculated from data in Figure 8 as described in the Methods section. The hydrophobic index (ClogP) for each compound was calculated by using the ChemDraw10.0 software (Cambridge Soft, UK) to allow deduction of the membrane permeability of each compound.

**Figure 5:**
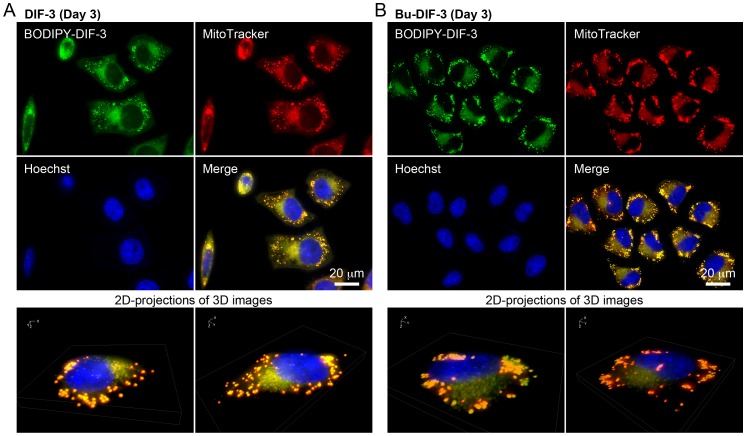
Cellular localization of BODIPY-DIF-3 in HeLa cells treated with DIF-3 or Bu-DIF-3. Cells were incubated for 3 days with DIF-3 (20 µM) (A) or Bu-DIF-3 (5 µM) (B), washed free of the additive, and further incubated for 0.5 h with BODIPY-DIF-3 (20 µM), Hoechst (0.1 µg/ml), and MitoTracker (0.1 µM). Cells were washed free of the additives and observed by using a high-magnification fluorescence microscope. Three-dimensional (3D) images were constructed, and two representative 2D-projections of the 3D images are shown. BODIPY-DIF-3 and MitoTracker co-localized to the mitochondria.

**Figure 6:**
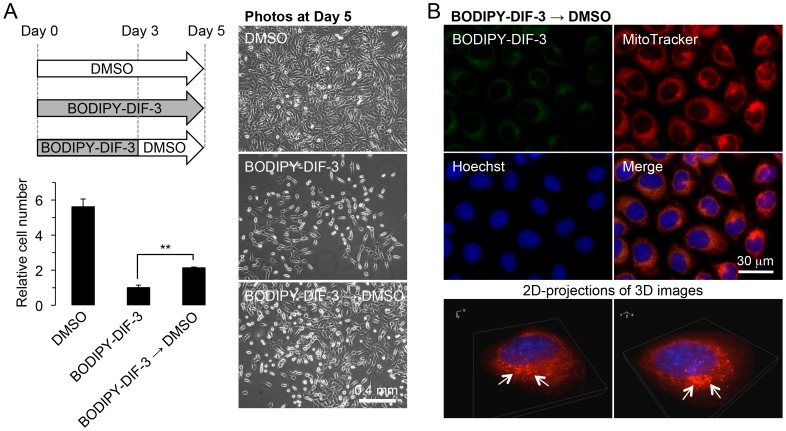
Recovery of HeLa cells treated with BODIPY-DIF-3. (A) As indicated in the scheme, control cells were incubated for 5 days with DMSO (0.2%), while the other sets of cells were incubated for 3 days with BODIPY-DIF-3 (20 µM), washed free of the additive, and further incubated for 2 days with BODIPY-DIF-3 (20 µM) or DMSO (0.2%). Cells were then observed by using a phase-contrast microscope, and relative cell number was assessed by the use of Alamar blue. Data are the mean value and SD (bars) of triplicates. Note that the relative cell number of DMSO control cells is presented for reference because the control cells had become confluent, at least in part, by Day 5. **, *P*<0.01. (B) Cells were incubated for 3 days with BODIPY-DIF-3 (20 µM), washed free of the additive, and further incubated for 2 days with DMSO (0.2%). The cells were then incubated for 0.5 h with Hoechst (0.1 µg/ml) and MitoTracker (0.1 µM), washed free of the additives, and observed by using a high-magnification fluorescence microscope. Three-dimensional (3D) images were constructed, and two representative 2D-projections of the 3D images are shown. Although some mitochondria remained slightly swollen (arrows), most of the mitochondria had recovered to their normal morphology and size. Mitochondria were weakly stained with BODIPY-DIF-3, which was virtually undetectable under the experimental conditions.

When HeLa cells were incubated for 3 days with DIF-3 (20 µM) and Bu-DIF-3 (5 µM), the mitochondria in all cells swelled greatly and stained with BODIPY-DIF-3 and MitoTracker (Figure 5). Taken together, these results suggest that DIF-3 and Bu-DIF-3, as well as BODIPY-DIF-3, target the mitochondria and induce swelling and dysfunction. Withdrawal of BOIDPY-DIF-3 at Day 3 induced recovery of cell growth rate and mitochondrial morphology in HeLa cells (Figure 6), indicating that the action of the drug is reversible.

### Analysis of Mitochondrial Morphology by using Electron Microscopy

**Figure 7:**
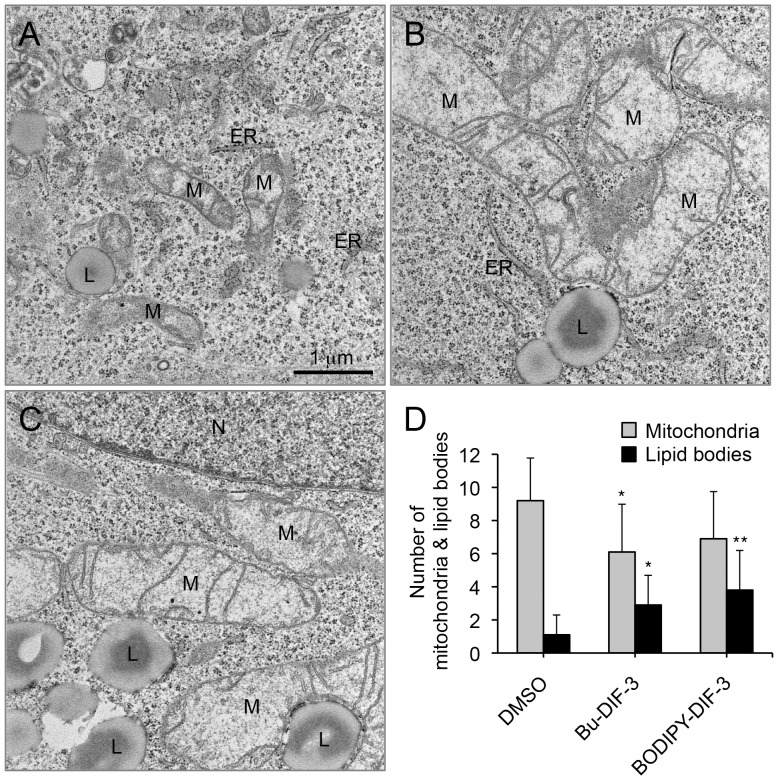
Electron microscopic analysis of HeLa cells treated with Bu-DIF-3 or BODIPY-DIF-3. (A–C) Cells were incubated for 3 days with DMSO (0.2%; A), Bu-DIF-3 (5 µM; B), or BODIPY-DIF-3 (20 µM; C). Cells were then treated as described in the Methods section and observed by using an electron microscope. Two representative images of three samples are shown. M, mitochondria. L, lipid body. N, nucleus. ER, endoplasmic reticulum. (D) The number of mitochondria and lipid bodies were counted in electron microscopic images that contained only the cytoplasmic region. Data are the mean values (the number of mitochondria or lipid bodies per image) and SD (bars) of ten separate images (n = 10). *, *P*<0.05; **, *P*<0.01 versus DMSO control.

We next investigated the effects of Bu-DIF-3 and BODIPY-DIF-3 on the morphology of organelles by using electron microscopy (Figure 7). As expected, the morphology of mitochondria was greatly altered when cells were incubated for 3 days with Bu-DIF-3 or BODIPY-DIF-3; cells treated with the compounds showed an increase in the number of swollen mitochondria (Figure 7A–C). In addition, Bu-DIF-3 and BODIPY-DIF-3 tended to cause a decrease in the total number of mitochondria (Figure 7D), possibly by inducing mitochondrial fusion and/or inhibiting their fission. It should be noted that many lipid bodies were observed in cells treated with Bu-DIF-3- and BODIPY-DIF-3 (Figure 7), suggesting that DIF-like molecules might affect lipid metabolism in some way. These results support the idea that DIF-like molecules are targeted to the mitochondria and could therefore affect the respiratory function of mitochondria.

### Effects of DIF-derivatives on O_2_ Consumption in Mitochondria

**Figure 8:**
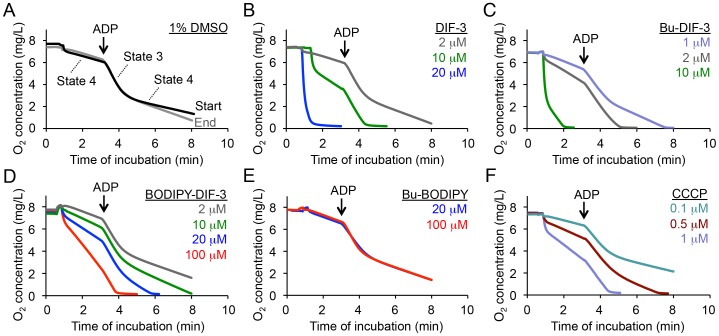
Effects of DIFs, BODIPY-conjugated compounds, or CCCP on oxygen consumption in mouse liver mitochondria. Mitochondria were prepared from mouse liver, and the effects of the indicated compounds on mitochondrial O_2_ consumption were assessed as described in the Methods section. After recording ‘State 4’ of the respiration reaction, ADP (200 µM: final concentration) was added to induce ‘State 3’. Mitochondrial reactivity did not alter over the course of the experiment from the starting (Start) to the ending (End) time points (A).

Based on the localization of DIF-3 derivatives to the mitochondria, we examined the effect of DIF-3 derivatives on mitochondrial O_2_ consumption in mouse liver mitochondria (Figure 8). In the presence of DMSO (vehicle), the O_2_ concentration in the culture medium deceased gradually at a constant rate (State 4), and the addition of ADP transiently accelerated the rate of O_2_ consumption (State 3) under these conditions (Figure 8A). By comparison, DIF-3, Bu-DIF-3, or BODIPY-DIF-3 at 1–100 µM dose-dependently promoted the basal rate of O_2_ consumption (Figure 8B–D), whereas the same concentrations of the non-bioactive reagent, Bu-BODIPY, had no effect on the rate of O_2_ consumption (Figure 8E). These results suggest that DIF-like molecules function as mitochondrial uncouplers and as such might suppress cell growth, at least in part, by disturbing mitochondrial activity.

### Effects of CCCP on Cell Growth and Mitochondrial Morphology

**Figure 9:**
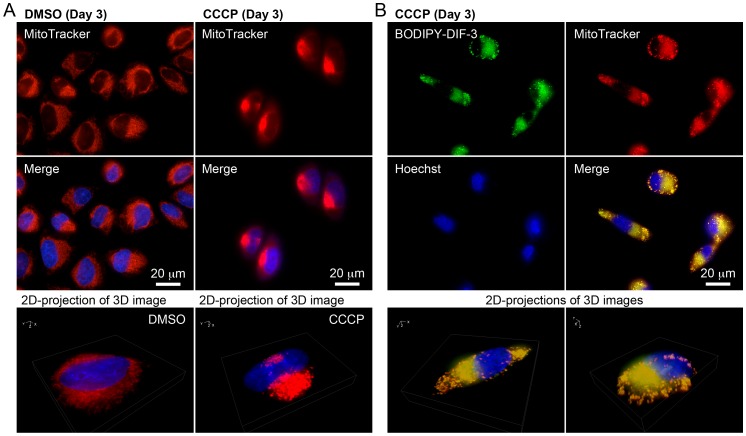
Cellular localization of BODIPY-DIF-3 in HeLa cells treated with CCCP. (A) Cells were incubated for 3 days with DMSO (0.2%) or CCCP (10 µM), washed free of the additive, and further incubated for 0.5 h with Hoechst (0.1 µg/ml) and MitoTracker (0.1 µM). Cells were washed free of the additives and observed by using a high-magnification fluorescence microscope. (B) Cells were incubated for 3 days with CCCP (10 µM), washed free of the additive, and further incubated for 0.5 h with BODIPY-DIF-3 (20 µM), Hoechst (0.1 µg/ml), and MitoTracker (0.1 µM). Cells were washed free of the additives and observed by using a high-magnification fluorescence microscope. Three-dimensional (3D) images were constructed, and two representative 2D-projections of the 3D images are shown. Note that CCCP induced mitochondrial swelling (A), which might be slightly enhanced by short-term incubation with BOIDPY-DIF-3 (B).

Since the DIF-3 derivatives appear to function by disturbing the respiratory function and morphology of mitochondria, we investigated the effects of CCCP, a mitochondrial uncoupler (proton-specific ionophore) (Figure 1A), for comparison. Cell growth and expression of cyclin D1 and D3 were significantly suppressed by CCCP at 10 µM in HeLa cells (Figure 3B, D, E), whereas azide (0.01%), an inhibitor of the transfer of electrons from cytochrome c to Complex IV, suppressed cell growth and the expression of cyclin D1 but not of cyclin D3; the mechanisms of the actions of azide and DIF-like molecules seem to be different at least in part. By comparison, CCCP at 0.1–1 µM promoted mitochondrial O_2_ consumption in a dose-dependent manner (Figure 8F). Mitochondrial swelling was induced in most HeLa cells after incubation for 3 days with CCCP at 10 µM; these mitochondria stained with BODIPY-DIF-3 and MitoTracker (Figure 9), which were similar to those in the cells incubated for 3 days with BODIPY-DIF-3 (Figure 4C, D), DIF-3 (Figure 5A), or Bu-DIF-3 (Figure 5B). It should be noted that both CCCP and BODIPY-DIF-3 dose-dependently suppressed cell growth in LM8 and 3T3-L1 cells (Figure S1A, S2A). Therefore, it is likely that DIF-like molecules function by disturbing mitochondrial activity, at least in part.

## Discussion

### DIFs as the Lead Compounds for Anti-tumor Drugs

*D. discoideum*, a cellular slime mold, is a well-studied model organism in the fields of cell and developmental biology. Cellular slime molds are soil microorganisms belonging to a kingdom different from fungi that produce many pharmacologically active compounds including polyketides. Thus, these organisms are expected to be a novel resource for lead compounds in the field of pharmacology and medicine [16]–[19]. Among the compounds reported so far, DIF-1 and DIF-3 (DIFs), members of the polyketide family, are the most promising and well-studied leads for the development of anti-tumor drugs [5]–[8], [13]. In mammals, DIFs affect the activity of protein kinases such as ERK and GSK-3β in some tumor cells [9]–[12] and arrest the cell cycle at the G0/1 phase by inhibiting the expression of cyclin D and the phosphorylation of retinoblastoma protein [12], [15]. We have also found that DIFs are direct inhibitors of calmodulin-dependent cyclic nucleotide phosphodiesterase (PDE1), suggesting that DIFs suppress cell growth, at least in part, by inhibiting PDE1 [14]. However, the inhibition of PDE1 may not fully explain the anti-tumor activities of DIFs [14] as there appears to be multiple DIF target molecules that may be affected indirectly rather than by direct interaction with the DIF. Thus, the precise mechanisms underlying the actions of DIF-like molecules in mammalian cells remain to be elucidated.

### Cellular Localization of DIF-like Molecules

Based on the chemical structures of DIFs and their solubility in both hexane and water, DIFs are thought to be membrane-permeable [3], but it is unknown whether they can penetrate the cell membrane and how they behave in mammalian cells. In the present study, we used the fluorescent DIF-3 derivative, BODIPY-DIF-3, to show for the first time that BODIPY-DIF-3 can penetrate the cell membrane and localize mainly to the mitochondria in HeLa, LM8, and 3T3-L1 cells (Figure 4, S1, S2). Careful observation suggested the presence of relatively BODIPY-DIF-3–dominant and MitoTracker–dominant mitochondria, possibly because the uptake of MitoTracker by mitochondria depends to some extent upon the mitochondrial membrane potential (see MitoTracker’s instructions), which would be disturbed by BODIPY-DIF-3. Therefore, when cells were stained first with MitoTracker, fixed with formalin, and then stained with BODIPY-DIF-3, MitoTracker and BODIPY-DIF-3 co-localized to the mitochondria more specifically (data not shown).

Since no organelles could be stained with Bu-BODIPY in living cells, formalin-fixed cells, or formalin/TritonX-100 treated cells (Figure 2B), it is likely that the presence of DIF-3 in BODIPY-DIF-3 is responsible for its membrane-permeability and specific localization to the mitochondria. Taken together with the findings that DIF-3 and Bu-DIF-3 as well as BODIPY-DIF-3 induced mitochondrial swelling in HeLa cells (Figure 4, 5), the results strongly suggest that DIF-3 and its derivatives can penetrate the cell membrane and localize to the mitochondria.

### Function of DIF-like Molecules in Mitochondria

DIF-1 is known to inhibit (uncouple) mitochondrial respiration with an IC_50_ value of 540 nM in *D. discoideum* [20], possibly because DIF-1 is a member of the phenol family and is a weak acid that behaves as an uncoupler (proton carrier across the mitochondrial membrane). However, since physiological concentrations of DIF-1 are thought to be in the range of 0.1–100 nM, the mitochondrial uncoupling activity of DIF-1 is unlikely to play a role in the development of *D. discoideum* [21]. Here, we showed with mammalian mitochondria that like CCCP, DIF-3, Bu-DIF-3, and BODIPY-DIF-3 promoted mitochondrial O_2_ consumption in a dose-dependent manner (Figure 8). In addition, the PC_100_ values of the DIF-3 derivatives for mitochondrial O_2_ consumption roughly correlated with their IC_50_ values (anti-proliferative activities) in the mammalian cells (Table 1). These results suggest that DIF-like molecules suppress cell growth, at least in part, by functioning as mitochondrial uncouplers, and also imply the presence of as-yet-unidentified target(s) for DIF-like molecules in mitochondria. In this regard, it was recently shown that DIF-1 binds to mitochondrial malate dehydrogenase (mMDH) and inhibits its activity [22]. However, because DIF-3 neither binds nor inhibits mMDH [22], it is likely that DIF-like molecules disturb mitochondrial function via target(s) other than mMDH. Note that Bu-DIF-3 and BODIPY-DIF-3 promoted lipid-body formation in cells treated with the compounds (Figure 7), suggesting that DIF-like molecules affect lipid metabolism possibly via mitochondrial dysfunction; the phenomenon will be elucidated in our next project.

As shown in Table 1, CCCP, DIF-3, Bu-DIF-3, and BODIPY-DIF-3 strongly suppress cell growth in HeLa cells and disturb mitochondrial O_2_ consumption with the indicated IC_50_ and PC_100_ values. The hydrophobic indexes (ClogP) of the compounds are also shown in Table 1, from which the membrane permeability of the compounds can be deduced to some extent. Since the chemical structure of CCCP is considerably different from that of DIF-3-related molecules (Figure 1A), it may not be appropriate to simply deduce and compare their behavior and functions according to their ClogP values. However, it is notable that the PC_100_ value for CCCP is much lower than its IC_50_ value, whereas the PC_100_ and IC_50_ values for DIF-3 are similar (Table 1). Nevertheless, the ClogP value of CCCP is comparable to that of DIF-3 (Table 1). Taken together, it is likely that the uncoupling of mitochondrial activity by CCCP is insufficient for growth inhibition by CCCP and/or that DIF-like molecules are more than mitochondrial uncouplers; e.g., DIF-1 and DIF-3 have been shown to be direct inhibitors of PDE1 [14].

Since mitochondria are the main energy producers in eukaryotic cells, mitochondrial poisons such as CCCP and azide are potent anti-proliferative and/or death-inducing agents in both cancer and non-cancer cells (present study) [23]–[27]. Furthermore, since mitochondria play a key role in cell death when mitochondrial membranes become permeabilized [28]–[30], mitochondria-directed chemotherapy is an emerging tool against cancer; in addition to known anticancer drugs, mitochondrial damaging agents such as CCCP are expected to be adjuvant drugs in chemotherapy to combat cancer [31], [32]. Accordingly, DIF-like molecules, as well as CCCP, may have potential as adjuvant drugs in cancer chemotherapy.

### Conclusions

We used a chemically synthesized fluorescent derivative of DIF-3 to show here, for the first time, that DIF-like molecules can penetrate the cell membrane, localize to the mitochondria, and promote mitochondrial oxygen consumption in mammalian cells. Our findings suggest that DIF-like molecules have the potential to suppress cell growth, at least in part, functioning as mitochondrial uncouplers to disturb mitochondrial activity. Although investigations into the role of DIF-like molecules in mitochondria have just begun, our findings provide significant insight into the function of DIF-like molecules in mammalian cells and set the foundation for further research. This is the first report showing the cellular localization and behavior of DIF-like molecules.

## Methods

### Reagents

MitoTracker® Red CMXRos (designated MitoTracker; Ex = 579, Em = 599 nm) and BODIPY®FL, SE (succinimidyl ester) (Ex = 505 nm, Em = 513 nm) were purchased from Invitrogen (currently, Life Technologies, Eugene, OR, USA). Hoechst 33342 (Ex = 352 nm, Em = 461) solution (1 mg/ml in H_2_O) and carbonyl cyanide *m*-chlorophenylhydrazone (CCCP) were obtained from Wako Pure Chemical Industries (Osaka, Japan). Rabbit anti-cyclin D1 (M-20), mouse anti-cyclin D2 (Was:14821A), and mouse anti-cyclin D3 (C-19) antibodies were purchased from Santa Cruz Biotechnology (Santa Cruz, CA, USA), BD Biosciences (San Jose, CA, USA), and Transduction Laboratories (C28620) (Lexington, KY, USA), respectively. Rabbit anti-GAPDH (14C10) antibody was purchased from Cell Signaling Technology (Beverly, MA, USA). Alkaline phosphatase–conjugated goat anti-mouse IgG and anti-rabbit IgG antibodies, nitroblue tetrazolium (NBT), and 5-bromo-4-chloro-3′-indolylphosphate (BCIP) were purchased from Promega (Madison, WI, USA). DIF-1, DIF-3, and Bu-DIF-3 were synthesized as described previously [13] and stored as solutions in dimethyl sulfoxide (DMSO) at –20°C.

### Cell Culture

Human cervical cancer HeLa cells (a kind gift from Dr. T. Oda, Gunma University, Japan) [33] and mouse 3T3-L1 fibroblasts (a kind gift from Dr. H. Shibata, Gunma University) [34] were grown and maintained at 37°C with 5% CO_2_ and 95% air in DMEM-FBS [Dulbecco’s Modified Eagle’s Medium containing 4,500 mg/l of glucose (Sigma, D5796) supplemented with 10% (v/v) heat-inactivated fetal bovine serum (FBS)]. Likewise, mouse osteosarcoma LM8 cells (a kind gift from Dr. F. Okajima, Gunma University) [35] were grown and maintained in MEM-α-FBS [Modified Eagle’s Medium-α (Wako Pure Chemical Industries, Ltd., Osaka, Japan) supplemented with 10% FBS]. All the media also contained 75 µg/ml penicillin and 50 µg/ml streptomycin.

### Synthesis of BODIPY-DIF-3

The synthesis of BODIPY-DIF-3 was a six-step process (Figure 1B) as described below.

#### Step 1: Synthesis of 5-(4-bromobutoxy)resorcinol

Potassium carbonate (7.17 g, 51.9 mmol) and 1,4-dibromobutane (3.15 ml, 26.4 mmol) were added to a solution of phloroglucinol (4.20 g, 25.9 mmol) in *N*,*N*-dimethylformamide (100 ml) at room temperature. The reaction mixture was stirred for 2 h at 50°C, diluted with 1 M hydrochloric acid (200 ml), and then extracted three times with ethyl acetate (250 ml). The combined organic layer was washed with water (200 ml) followed by saturated sodium chloride solution (200 ml), dried over sodium sulfate, and evaporated under reduced pressure. The residue was chromatographed over a silica gel column with a hexane-ethyl acetate (2∶1) solvent system to give 5-(4-bromobutoxy)resorcinol (1.94 g, 7.43 mmol).

#### Step 2: Synthesis of 1-(4-(4-bromobutoxy)-2,6-dihydroxyphenyl)hexan-1-one

Hexanoyl chloride (0.720 ml, 5.15 mmol) and aluminium chloride (1.34 g, 10.1 mmol) were added to a solution of 5-(4-bromobutoxy)resorcinol (1.31 g, 5.03 mmol) in dichloromethane (30 ml). The reaction mixture was stirred for 3 h at room temperature, diluted with water (100 ml), and then extracted with ethyl acetate (150 ml) three times. The combined organic layer was washed with saturated sodium bicarbonate solution (150 ml) followed by saturated sodium chloride solution (150 ml), dried over sodium sulfate, and evaporated under reduced pressure. The residue was chromatographed over a silica gel column with a hexane-ethyl acetate (9∶1) solvent system to give 1-(4-(4-bromobutoxy)-2,6-dihydroxyphenyl)hexan-1-one (0.910 mg, 2.53 mmol).

#### Step 3: Synthesis of 1-(4-(4-bromobutoxy)-3-chloro-2,6-dihydroxyphenyl)hexan-1-one

Sulfuryl chloride (342 mg, 2.53 mmol) was added to a solution of 1-(4-(4-bromobutoxy)-2,6-dihydroxyphenyl)hexan-1-one (910 mg, 2.53 mmol) in chloroform-ethanol (49∶1) (25 ml). The reaction mixture was stirred for 1 h at room temperature, and then evaporated under reduced pressure. The residue was chromatographed over a silica gel column with a hexane-ethyl acetate (9∶1) solvent system to give 1-(4-(4-bromobutoxy)-3-chloro-2,6-dihydroxyphenyl)hexan-1-one (899 mg, 2.28 mmol).

#### Step 4: Synthesis of 1-(4-(4-azidobutoxy)-3-chloro-2,6-dihydroxyphenyl)hexan-1-one

Sodium azide (223 mg, 3.42 mmol) was added to a solution of 1-(4-(4-bromobutoxy)-3-chloro-2,6-dihydroxyphenyl)hexan-1-one (337 mg, 0.855 mmol) in *N*,*N*-dimethylformamide (8 ml) at room temperature. The reaction mix was stirred for 3 h, and then diluted with water (30 ml) and extracted with ethyl acetate (40 ml) three times. The combined organic layer was washed with water (40 ml) followed by saturated sodium chloride solution (40 ml), dried over sodium sulfate, and evaporated under reduced pressure. The residue was chromatographed over a silica gel column with a hexane-ethyl acetate (4∶1) solvent system to give 1-(4-(4-azidobutoxy)-3-chloro-2,6-dihydroxyphenyl)hexan-1-one (302 mg, 0.849 mmol).

#### Step 5: Synthesis of 1-(4-(4-aminobutoxy)-3-chloro-2,6-dihydroxyphenyl)hexan-1-one hydrochloride

Palladium on carbon (5% w/w, 2.0 mg) was added to a solution of 1-(4-(4-azidobutoxy)-3-chloro-2,6-dihydroxyphenyl)hexan-1-one (52 mg, 0.145 mmol) in 3% (w/v) hydrochloride methanol solution (3 ml) at room temperature. The reaction mixture was stirred for 1 h under an atmosphere of hydrogen, and then filtered through a pad made of Celite (Nacalai Tesque, Inc., Kyoto, Japan). The Celite pad was washed with methanol, and the filtrate was evaporated under reduced pressure. The residue was chromatographed over a silica gel column with a chloroform-methanol (4∶1) solvent system to give 1-(4-(4-aminobutoxy)-3-chloro-2,6-dihydroxyphenyl)hexan-1-one hydrochloride (52 mg, 0.142 mmol).

#### Step 6: Synthesis of BODIPY-DIF-3

1-(4-(4-Aminobutoxy)-3-chloro-2,6-dihydroxyphenyl)hexan-1-one hydrochloride (7.6 mg, 20.7 µM) and triethylamine (20 µl) were added to a solution of BODIPY® FL, SE (1.9 mg, 4.8 µmol) in tetrahydrofurane (1 ml) at room temperature in the dark. The reaction mixture was stirred for 5 h, diluted with 0.2 M hydrochloric acid (5 ml) and then extracted three times with ethyl acetate (10 ml). The residue was subjected to recycling preparative HPLC with a JAIGEL-GS-310 column (internal diameter 20 mm, length 500 mm, Japan Analytical Industry Co., Ltd.) using ethyl acetate as the solvent to give BODIPY-DIF-3 (1.9 mg, 4.8 µmol). Analytical data for BODIPY-DIF-3 was as follows: ^1^H NMR (400 MHz, CDCl_3_) δ 7.07 (1H, s), 6.83 (1H, d, *J* = 4.0 Hz), 6.25 (1H, d, *J* = 4.0 Hz), 6.13 (1H, s), 6.07 (1H, s), 5.93 (1H, br.s), 3.99 (2H, t, *J* = 7.4 Hz), 3.32 (2H, q, *J* = 6.6 Hz), 3.27 (2H, t, *J* = 7.3 Hz), 3.09 (2H, t, *J* = 7.4 Hz), 2.69 (2H, t, *J* = 7.3 Hz), 2.57 (3H, s), 2.24 (3H, s), 1.68 (2H, quint, *J* = 7.4 Hz), 1.54–1.63 (4H, m), 1.30–1.39 (4H, m), 0.92 (3H, t, *J* = 7.1 Hz); HRFABMS *m/z* 584.2517 [M-F]^+^ (584.2499 calculated for C_30_H_37_N_3_O_5_B^35^ClF).

### Synthesis of Bu-BODIPY

As described briefly in Figure 1B, 1-butylamine (20 µl) and triethylamine (20 µl) were added to a solution of BODIPY® FL, SE (1.9 mg, 4.8 µmol) in *N*,*N*-dimethylformamide (1 ml) at room temperature in the dark. The reaction mixture was stirred for 10 h, diluted with 0.2 M hydrochloric acid (5 ml), and then extracted three times with ethyl acetate (10 ml). The residue was chromatographed over a silica gel column with a chloroform-methanol (99∶1) solvent system to give Bu-BODIPY (1.6 mg, 4.7 µmol). Analytical data for Bu-BODIPY was as follows:^ 1^H NMR (400 MHz, CDCl_3_) δ 7.09 (1H, s), 6.89 (1H, d, *J* = 3.9 Hz), 6.30 (1H, d, *J* = 3.9 Hz), 6.13 (1H, s), 5.67 (1H, br.s), 3.27 (2H, t, *J* = 7.4 Hz), 3.20 (2H, q, *J* = 6.9 Hz), 2.62 (2H, t, *J* = 7.4 Hz), 2.57 (3H, s), 2.26 (3H, s), 1.40 (2H, quint, *J* = 6.9 Hz), 1.23–1.29 (2H, m), 0.87 (3H, t, *J* = 7.3 Hz); HRFABMS *m/z* 322.0605 [M-F]^+^ (328.1996 calculated for C_18_H_24_N_3_OBF). BODIPY-conjugated compounds were stored as solutions in DMSO (dimethyl sulfoxide) at –20°C.

### Cell Growth Assay

HeLa, LM8, and 3T3-L1 cells were cultured for 3 days at 2.5–5×10^3^ cells/well in 12-well plates with each well filled with 1 ml of DMEM-FBS (for HeLa and 3T3-L1) or MEM-α-FBS (for LM8) together with drugs at various concentrations. Then, the incubation media were discarded and the cells were washed with 1 ml of PBS(–) (20 mM phosphate buffered saline, pH 7.4) and incubated with 1 ml of fresh DMEM-FBS (HeLa and 3T3-L1) or MEM-α-FBS (LM8) containing 5% (v/v) of Alamar blue (a cell number indicator; Wako Pure Chemical Industries, Osaka, Japan) until the color changed. The relative cell number was assessed by measuring absorbance at 570 nm (reference at 595 nm) as described previously [8], [14]; DIF-related compounds and BODIPY-conjugated compounds hardly disturbed the cell number assessment with Alamar blue (data not shown), probably because the additive compounds were washed out when cell number was assessed. For determination of the 50% inhibitory concentration (IC_50_) of each drug, the cells were cultured for 3 days in the presence of each drug at various concentrations. Relative cell numbers were determined for each concentration for each drug and the IC_50_ was determined from the dose-response curves.

In the assay of the effect of withdrawal of BODIPY-DIF-3 on cell growth (Figure 6A), HeLa cells were incubated for 3 days with 2 ml of DMEM-FBS containing 20 µM BODIPY-DIF-3 in 35-mm tissue culture dishes (Becton Dickinson, Franklin Lakes, NJ, USA) (5×10^3^ cells/dish), washed 2 times with 2 ml of PBS(–), and further incubated for 2 days with 2 ml of DMEM-FBS containing 0.2% DMSO or 20 µM BODIPY-DIF-3; meanwhile, control cells were incubated for 5 days with 2 ml of DMEM-FBS containing 0.2% DMSO. Relative cell number was assessed as described above, and all experiments were performed in triplicate.

### Western Blotting

HeLa cells were incubated for 20 h with 1 ml of DMEM-FBS containing the indicated additives in 12-well plates (2–4×10^5^ cells/well), washed twice with PBS(–), harvested by adding 100–200 µl of an SDS-sample buffer solution (in proportion to cell density: relative cell number), sonicated (heated), and used for SDS-PAGE. Western blot transfer and immunoblotting were performed as described previously [36], by using a primary antibody for cyclin D1, cyclin D2, cyclin D3, or GAPDH, and a second antibody, an alkaline phosphatase-conjugated anti-mouse or anti-rabbit IgG antibody. Color development (visualization of the protein bands) was performed in an alkaline buffer (100 mM Tris-HCl, pH 9.5, 100 mM NaCl, 5 mM MgCl_2_) containing NBT (125 µg/ml) and BCIP (62.5 µg/ml). Visualized protein bands were then digitized and quantified by using Adobe Photoshop CS4 (version 11.0) (Adobe, San Jose, CA, USA) and ImageJ Software (version 1.4) (http://rsb.info.nih.gov/ij/).

### Formalin Fixation and Triton X-100 Treatment

HeLa cells were cultured for 1–2 days with 2 ml DMEM-FBS in 35-mm tissue culture dishes (Becton Dickinson), washed twice with PBS(–), and then fixed for 15–20 min at room temperature with 2 ml of 3.7% (v/v) formaldehyde in PBS(–). The cells were then washed twice with PBS(–) and used for staining with the fluorescent probes. Alternatively, the fixed cells were treated for 15–20 min with 2 ml of 0.1% (w/v) Triton X-100 in PBS(–), washed twice with PBS(–), and used for staining with the fluorescent probes.

### Phase-contrast and Fluorescence Microscopy

For low magnification observations, living cells, formalin-fixed cells, or Triton X-treated cells were incubated with 2 ml of DMEM-FBS (HeLa and 3T3-L1) or MEM-α-FBS (LM8) containing 20 µM Bu-BODIPY, or BODIPY-DIF-3, and in some cases, Hoechst (1 µg/ml) and MitoTracker (0.1 µM) in 35-mm tissue culture dishes (Becton Dickinson). The cells were washed twice with PBS(–) and submerged in 2 ml of PBS(–) (for fixed-cells) or a Hepes buffer (0.1% bovine serum albumin, 137.5 mM NaCl, 5 mM KCl, 2.5 mM CaCl_2_, 0.8 mM MgCl_2_, 5.5 mM glucose, 0.6 mM NaHCO_3_, 20 mM Hepes-NaOH pH 7.4) (for living cells). The cells were observed at room temperature with a Leica DM IRB fluorescent microscope (Wetzlar, Germany), and digitized images were treated with the Leica Application Suite (version 3.3.0). For time-course observation of the behavior (permeability) of BODIPY-DIF-3, cells were incubated in the presence of 20 µM BODIPY-DIF-3 in Hepes buffer and observed at the indicated time points. At 30 min of incubation, the cells were washed twice with PBS(–), submerged in 2 ml of Hepes buffer, and observed microscopically.

For high magnification multi-color imaging, cells were incubated for the appropriate times with 2 ml of DMEM-FBS (HeLa and 3T3-L1) or MEM-α-FBS (LM8) containing various compounds in 35-mm plastic dishes (tissue culture treated µ-Dish; ibidi, Martinsried, Germany). The cells were washed twice with 2 ml of PBS(–), submerged in 2 ml of Hepes buffer (pH 7.4), and observed at room temperature with a Keyence BZ-9000 fluorescence microscope (Osaka, Japan) equipped with an oil immersion 100× lens (CFI Plan Apo VC100XH) and multi-filters that can distinguish four fluorescent probes simultaneously. Original digitized images of z-stack sections were taken at 0.4- µm intervals, which were then treated (haze-reduced) with the Keyence BZ analyzer software (for deconvolution fluorescence imaging), and compiled into 3D images; when 3D images were constructed, nonlinear adjustment was performed to obtain clear (high contrast) images without haze. All color images are presented in pseudo colors.

### Electron Microscopy

HeLa cells were incubated for 3 days with 2 ml DMEM-FBS containing 0.2% DMSO and 5 µM Bu-DIF-3 or 20 µM BODIPY-DIF-3 in 35-mm plastic dishes at 2×10^4^ cells/dish. The cells were fixed at 4°C for 30 min with PB (0.1 M phosphate buffer, pH 7.4) containing 2% paraformaldehyde and 2% glutaraldehyde and then with PB containing 2% glutaraldehyde at 4°C overnight. The cell samples were rinsed 3 times with PB for 30 min each, followed by post fixation with 2% OsO_4_ in PB at 4°C for 1 h. The samples were dehydrated through a graded ethanol (EtOH) series starting with 50% and 70% EtOH for 5 min each at 4°C, then 90% EtOH for 5 min at room temperature, followed by three changes of 100% EtOH for 5 min each at room temperature. The samples were then transferred to a resin (Quetol-812; Nisshin EM Co., Tokyo, Japan) and polymerized at 60°C for 48 h. Ultra-thin (70 nm) sections were cut with an ultramicrotome (ULTRACUT UCT; Leica, Wetzlar, Germany), collected onto copper grids, stained with 2% uranyl acetate at room temperature for 15 min, and then rinsed with distilled water. The sections were then stained with Lead stain solution (Sigma-Aldrich) at room temperature for 3 min. The sections were observed under a transmission electron microscope (JEM-1200Ex; JEOL Ltd., Tokyo, Japan) at an acceleration voltage of 80 kV. Digital images (2048×2048 pixels) were taken with a charge-coupled device camera (VELETA; Olympus Soft Imaging Solutions GmbH, Tokyo, Japan).

### Preparation of Mitochondria-enriched Fraction

Mitochondria were isolated from mouse liver (ICR; 7–10 week-old females) by differential centrifugation as described previously [37]. Cells were homogenized in a Potter glass homogenizer with H-Buffer (250 mM sucrose, 10 mM Tris-HCl pH 7.4), and centrifuged at 800*g* for 1 min at 4°C. The supernatant was centrifuged at 6,000*g* for 5 min, and the resulting pellet, the crude mitochondrial fraction, was suspended in H-Buffer. The suspension was layered over a discontinuous sucrose gradient consisting of 1.1 M and 1.6 M sucrose in a Tris buffer (10 mM Tris-HCl pH 7.4), and centrifuged for 3 h at 90,000*g* at 4°C. The interface was collected in Tris buffer and centrifuged at 6,000*g* for 5 min. The resulting pellets were suspended in Tris buffer and used for experiments after confirming the presence of the mitochondrial marker cytochrome c.

### Oxygen Consumption Analysis

Mitochondrial oxygen consumption was determined using a Clark-type oxygen electrode (Strathkelvin Instruments Ltd., North Lanarkshire, Scotland) as described [38], [39]. The mitochondria-enriched fraction was incubated at 30°C in oxygen measurement buffer (225 mM mannitol, 75 mM sucrose, 10 mM KCl, 0.1 mM EDTA, 3 mM phosphate, 5 mM succinate, 5 mM glutamate, and 20 mM Tris-HCl pH 7.4) in the presence or absence of various concentrations of the DIF-like molecules or CCCP. After recording ‘State 4’ of the respiration reaction, an aliquot of ADP was added to a final concentration of 200 µM to induce ‘State 3’. The PC_100_ (100% promoting concentration) of each additive was defined as the drug concentration that doubled the basal rate of oxygen consumption during State 3, which was calculated from the dose-response plot for the rate of oxygen consumption (Figure 8).

## Supporting Information

Figure S1 **Effects of BODIPY-DIF-3 and CCCP on cell growth, and cellular localization of BODIPY-DIF-3 in LM8 cells.** (A) Cells were incubated for 4 days with 5–20 µM of BODIPY-DIF-3 (closed circles) or 2–10 µM of CCCP (closed triangles), and relative cell number was assessed by the use of Alamar blue. Data are the mean values and SD (bars) of three independent experiments. (B) Cells were incubated for 0.5 h or 3 days with 20 µM of BODIPY-DIF-3, washed free of the additive, and observed microscopically. (C) Cells were incubated for 0.5 h with BODIPY-DIF-3 (20 µM), Hoechst (0.1 µg/ml), and MitoTracker (0.1 µM), washed free of the additives, and observed by using a high-magnification fluorescence microscope. (D) Cells were incubated for 3 days with BODIPY-DIF-3 (20 µM) and then for 0.5 h with Hoechst (0.1 µg/ml) and MitoTracker (0.1 µM). Cells were washed free of the additives and observed by using a high-magnification fluorescence microscope.
(TIF)

Figure S2 **Effects of BODIPY-DIF-3 and CCCP on cell growth, and cellular localization of BODIPY-DIF-3 in 3T3-L1 cells.** (A) Cells were incubated for 4 days with 5–20 µM of BODIPY-DIF-3 (closed circles) or 2–10 µM of CCCP (closed triangles), and relative cell number was assessed by the use of Alamar blue. Data are the mean values and SD (bars) of three independent experiments. (B) Cells were incubated for 0.5 h or 3 days with 20 µM of BODIPY-DIF-3, washed free of the additive, and observed microscopically. (C) Cells were incubated for 0.5 h with BODIPY-DIF-3 (20 µM), Hoechst (0.1 µg/ml), and MitoTracker (0.1 µM), washed free of the additives, and observed by using a high-magnification fluorescence microscope. (D) Cells were incubated for 3 days with BODIPY-DIF-3 (20 µM) and then for 0.5 h with Hoechst (0.1 µg/ml) and MitoTracker (0.1 µM). Cells were washed free of the additives and observed by using a high-magnification fluorescence microscope.
(TIF)
