# Correction: Mitochondria Are the Target Organelle of Differentiation-Inducing Factor-3, an Anti-Tumor Agent Isolated from *Dictyostelium discoideum*

**DOI:** 10.1371/annotation/7a1f1670-ee25-436a-867c-3d5e160ac6cb

**Published:** 2013-08-21

**Authors:** Yuzuru Kubohara, Haruhisa Kikuchi, Yusuke Matsuo, Yoshiteru Oshima, Yoshimi Homma

There was an error in capitalization in the article title.

The correct title is: Mitochondria Are the Target Organelle of Differentiation-Inducing Factor-3, an Anti-Tumor Agent Isolated from *Dictyostelium discoideum*

The correct citation is: Kubohara Y, Kikuchi H, Matsuo Y, Oshima Y, Homma Y (2013) Mitochondria Are the Target Organelle of Differentiation-Inducing Factor-3, an Anti-Tumor Agent Isolated from *Dictyostelium discoideum*. PLoS ONE 8(8): e72118. doi:10.1371/journal.pone.0072118 

