# Supplementary figures and images for: Mitochondria Are the Target Organelle of Differentiation-Inducing Factor-3, an Anti-Tumor Agent Isolated from Dictyostelium Discoideum
Source: PLoS One. 2013 Aug 15;8(8):e72118. doi: 10.1371/journal.pone.0072118 (PMC3744471; doi:10.1371/journal.pone.0072118)

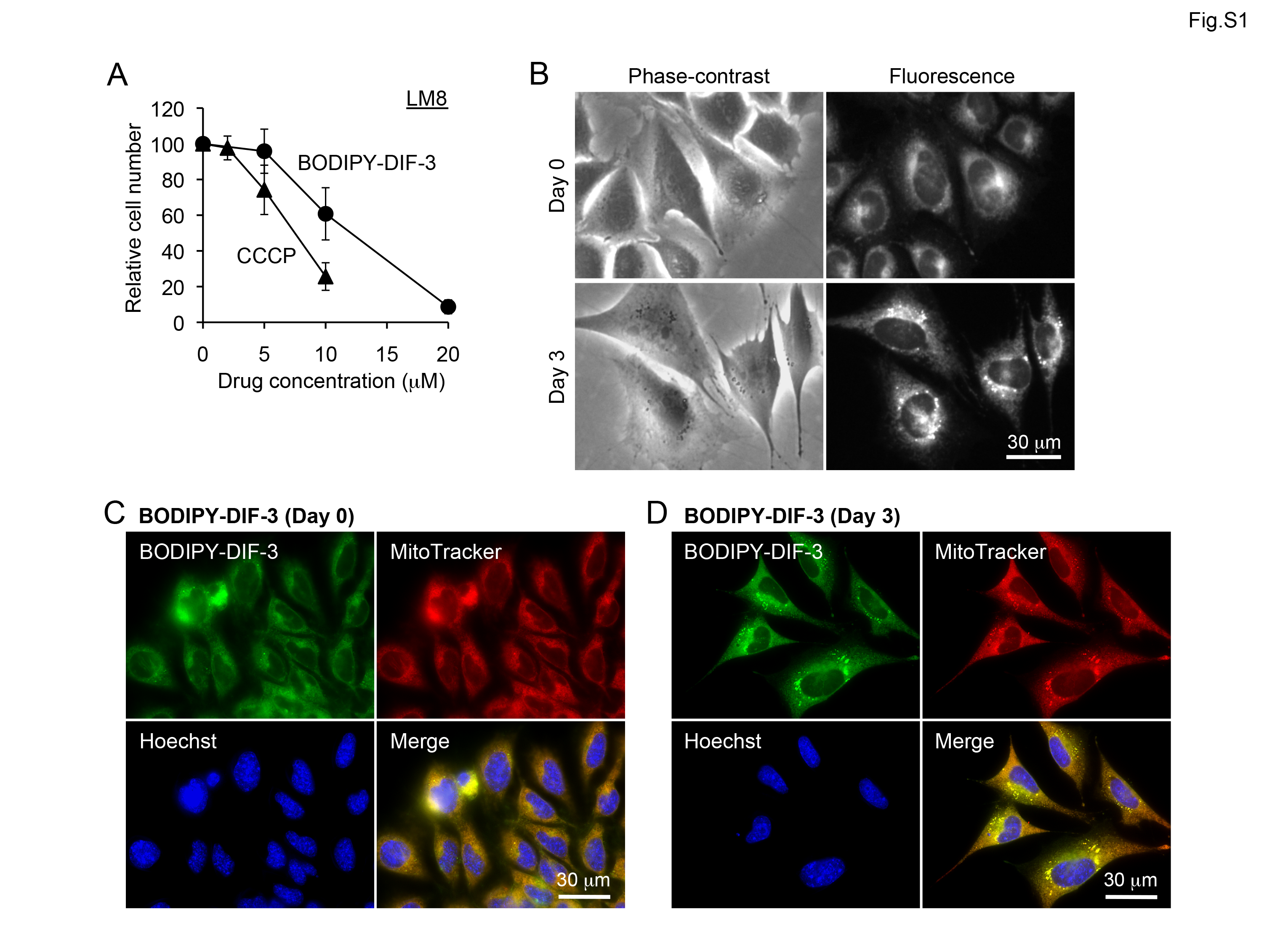

Supplement: Figure S1 — Effects of BODIPY-DIF-3 and CCCP on cell growth, and cellular localization of BODIPY-DIF-3 in LM8 cells. (A) Cells were incubated for 4 days with 5–20 µM of BODIPY-DIF-3 (closed circles) or 2–10 µM of CCCP (closed triangles), and relative cell number was assessed by the use of Alamar blue. Data are the mean values and SD (bars) of three independent experiments. (B) Cells were incubated for 0.5 h or 3 days with 20 µM of BODIPY-DIF-3, washed free of the additive, and observed microscopically. (C) Cells were incubated for 0.5 h with BODIPY-DIF-3 (20 µM), Hoechst (0.1 µg/ml), and MitoTracker (0.1 µM), washed free of the additives, and observed by using a high-magnification fluorescence microscope. (D) Cells were incubated for 3 days with BODIPY-DIF-3 (20 µM) and then for 0.5 h with Hoechst (0.1 µg/ml) and MitoTracker (0.1 µM). Cells were washed free of the additives and observed by using a high-magnification fluorescence microscope. (TIF) [file pone.0072118.s001.tif]

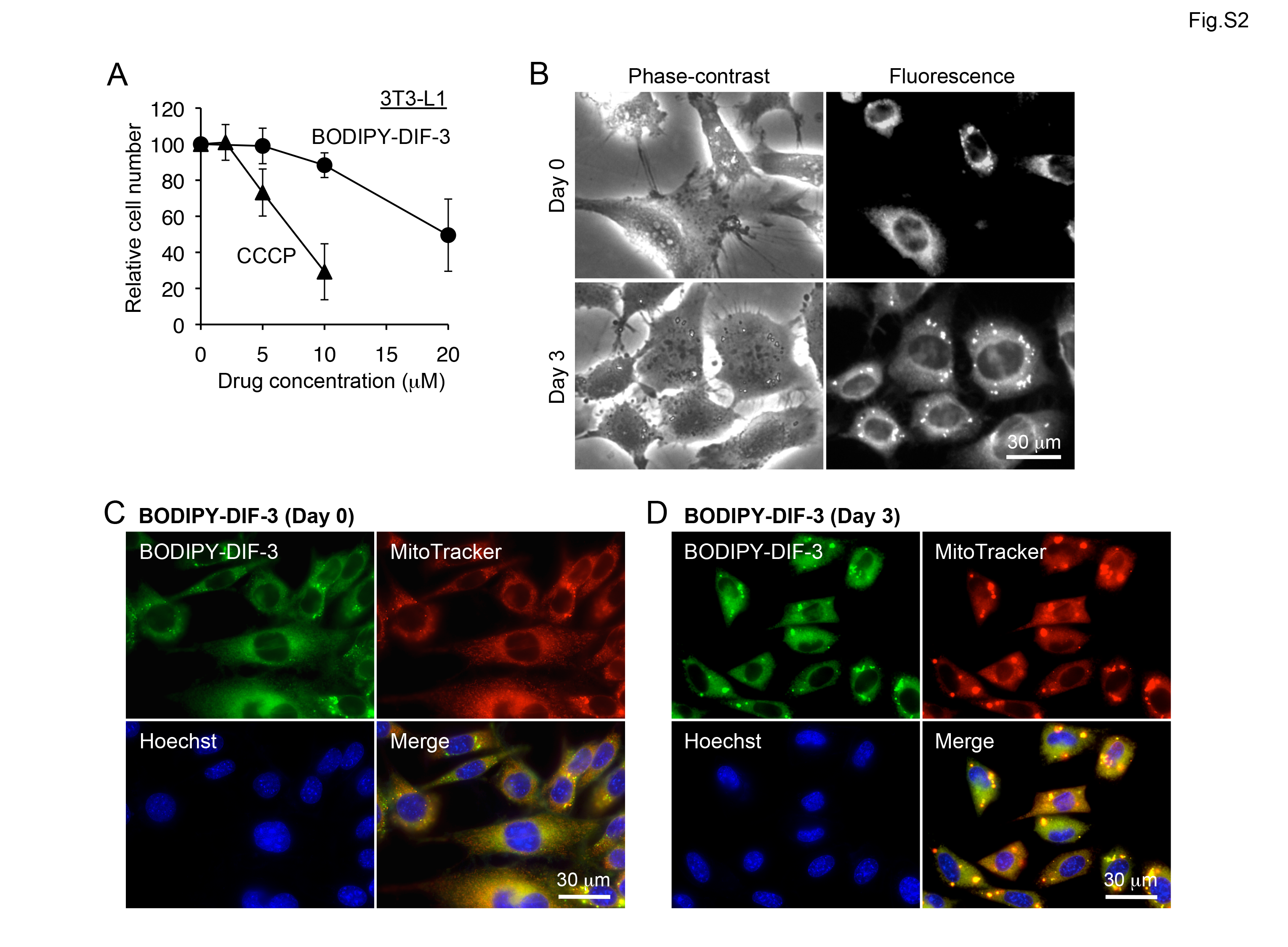

Supplement: Figure S2 — Effects of BODIPY-DIF-3 and CCCP on cell growth, and cellular localization of BODIPY-DIF-3 in 3T3-L1 cells. (A) Cells were incubated for 4 days with 5–20 µM of BODIPY-DIF-3 (closed circles) or 2–10 µM of CCCP (closed triangles), and relative cell number was assessed by the use of Alamar blue. Data are the mean values and SD (bars) of three independent experiments. (B) Cells were incubated for 0.5 h or 3 days with 20 µM of BODIPY-DIF-3, washed free of the additive, and observed microscopically. (C) Cells were incubated for 0.5 h with BODIPY-DIF-3 (20 µM), Hoechst (0.1 µg/ml), and MitoTracker (0.1 µM), washed free of the additives, and observed by using a high-magnification fluorescence microscope. (D) Cells were incubated for 3 days with BODIPY-DIF-3 (20 µM) and then for 0.5 h with Hoechst (0.1 µg/ml) and MitoTracker (0.1 µM). Cells were washed free of the additives and observed by using a high-magnification fluorescence microscope. (TIF) [file pone.0072118.s002.tif]
